# Innate Immunity Modulation by the IL-33/ST2 System in Intestinal Mucosa

**DOI:** 10.1155/2013/142492

**Published:** 2012-12-04

**Authors:** Marina García-Miguel, M. Julieta González, Rodrigo Quera, Marcela A. Hermoso

**Affiliations:** ^1^Disciplinary Program of Immunology, Institute of Biomedical Sciences, Faculty of Medicine, University of Chile, Santiago, Chile; ^2^Cell and Molecular Biology Program, Biomedical Sciences Institute, Faculty of Medicine, University of Chile, Santiago, Chile; ^3^Gastroenterology Unit, Las Condes Clinic, Santiago, Chile

## Abstract

Innate immunity prevents pathogens from entering and spreading within the body. This function is especially important in the gastrointestinal tract and skin, as these organs have a large surface contact area with the outside environment. In the intestine, luminal commensal bacteria are necessary for adequate food digestion and play a crucial role in tolerance to benign antigens. Immune system damage can create an intestinal inflammatory response, leading to chronic disease including inflammatory bowel diseases (IBD). Ulcerative colitis (UC) is an IBD of unknown etiology with increasing worldwide prevalence. In the intestinal mucosa of UC patients, there is an imbalance in the IL-33/ST2 axis, an important modulator of the innate immune response. This paper reviews the role of the IL-33/ST2 system in innate immunity of the intestinal mucosa and its importance in inflammatory bowel diseases, especially ulcerative colitis.

## 1. Introduction

The gastrointestinal tract is a cavity that begins in the mouth and extends to the anus. It is a part of the digestive system, and its primary function is to physically and chemically digest food for nutrient capture by cells. To achieve these functions, the presence of commensal bacteria or microflora is necessary. The innate immune elements of the intestinal mucosa seem to have the ability to recognize antigens from the microbiota as well as from food proteins, avoiding an inflammatory response to these benign antigens. This process is referred to as an intestinal tolerance. If the process is disrupted, the immune response is activated. This aberrant immune response contributes to the physiopathology of diseases such as food allergies, celiac disease (gluten immune response), and inflammatory bowel diseases (IBD) including ulcerative colitis (UC) and Crohn's disease (CD). UC is characterized by inflammation and ulceration of intestinal mucosa in the colon and rectum, while CD involves complete transmural compromise of the gastrointestinal tract.

In recent years, the axis of interleukin-33 (IL-33) and its receptor ST2 has been recognized as crucial to the homeostasis of the epithelial inflammatory response, including within the intestinal epithelium. Patients with UC show increased serum ST2 as well as higher IL-33 levels in the intestinal mucosa [1]. Furthermore, serum ST2 level correlates with disease severity and, therefore, might be a biomarker of disease activity [2].

In this paper, we will evaluate the role of the IL-33/ST2 system in innate immunity of the intestinal mucosa and IBD, especially UC. 

## 2. Intestinal Innate Immunity and Its Alteration in Ulcerative Colitis

**Figure 1:**
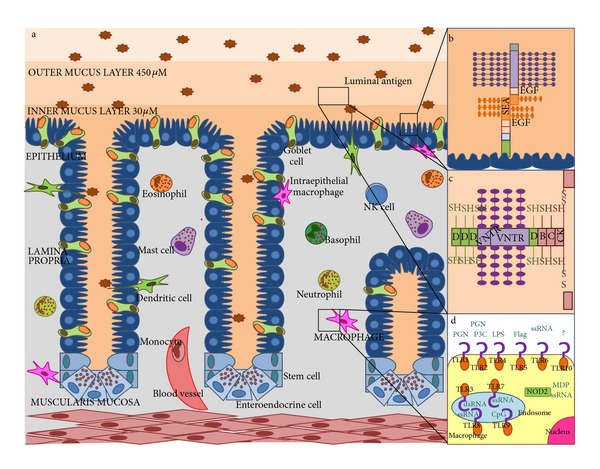
Components of intestinal innate immune system. (a) Lines of intestinal innate defense: mucus layer (outer and inner mucus layer), epithelium and *lamina propria *with different cells. (b) Representation of a surface-anchored mucin protein. (c) Representation of an oligomeric secreted mucin protein. (d) Cellular distribution of TLRs and NOD receptors and their ligands in an innate immune cell.

The intestinal innate immune system involves three lines of defense: the mucus layer, epithelium, and *lamina propria *(Figure 1(a)). When these barriers are damaged, immunological tolerance may be affected. 

### 2.1. Mucus as a First Line of Intestinal Defense

The gastrointestinal tract is covered by a layer of mucus that protects the epithelium from luminal antigens and provides lubrication to advance the bolus. In the stomach and colon, mucus consists of two layers: one layer well adhered to the epithelium, about 30 *μ*m thick, and another easily removable layer, about 450 *μ*m thick. Only the second layer is present in other areas, such as the small intestine. Mucus varies in quantity and in protein composition in different areas of the gastrointestinal tract. Mucus is most abundant in the colon, due to higher bacterial content. The major constitutive proteins are mucins (MUC), with diverse isotypes in different portions of the gastrointestinal tract. In the small intestine, the most abundant mucins are MUC2, MUC3 and MUC6, while, in the colon, the principal protein is MUC2. However, gastrointestinal tract cells also express other mucins, such as MUC1, MUC3, MUC4, MUC11 and MUC12 [3].

Different MUC isoforms result in greater or lesser adhesion of the mucus layer to the epithelium. Some mucins such as MUC1, adhere firmly to the epithelial layer. These proteins are expressed primarily in enterocytes (Figure 1(b)). This mucus barrier is important in halting bacteria invasion, as shown in MUC1-deficient mice (MUC1^−/−^), which are unable to eliminate *Campylobacter jejuni* and succumb to a systemic infection [4]. On the other hand, other MUCs are synthesized and secreted by goblet cells that constitute the easily removable mucus layer (Figure 1(c)) [5–7].

Goblet cells exhibit a characteristic morphology that resembles an elongated cup, due to a large apical theca that contains the mucin granules. Goblet cell differentiation is partially dependent on the presence of bacteria. In germ-free mice born and bred in aseptic conditions, goblet cells are smaller and reduced in number as compared to wild-type mice [8]. In addition, goblet cells are more abundant in the normal colon than in the duodenum, due to greater amounts of microbiota in this region [9]. Goblet cell differentiation is determined by the NOTCH signaling pathway and expression of transcription factors HATH1 and SPDEF [10–12].

MUC2 is the most abundant protein in the colon. It has a mass of 540 kDa and is composed of over 85% glycosidic residues. In terms of the aminoacidic structure of MUC2, the core domains are poorly conserved and rich in tandem repeats. Large amounts of proline, threonine, and serine residues make these domains susceptible to O-glycosylations [13–15], as well as the incorporation of sulfate and sialic groups, providing the ability to bind water and pathogens. The absence of MUC2 biosynthetic enzymes that catalyze the first O-glycosylation in the Golgi apparatus (GalNAc) promotes spontaneous development of colitis in mice [16]. Additionally, MUC2 glycosylations can be metabolized by intestinal commensal or pathogenic bacteria, serving as an energy source [17, 18], suggesting a role in intestinal microbiota selection. 

MUC2 has four D prepro-von Willebrand factor domains, three in the amino- and one in the carboxyterminal. These protein regions, such as the cystein-bond domain in the carboxyl terminus, are highly conserved and rich in cystein residues, enabling disulphide bridge formation and protein oligomerization, and increasing mucus viscosity [19–24].

MUC2^−/−^ animals spontaneously develop colitis with inflammation of the colon mucosa, have diarrhea containing neutrophils, and develop rectal prolapse or even cancer, similar to human UC [25]. Furthermore, wild-type mice treated with dodecyl dextran sulphate (DSS), an agent that reduces mucus layer thickness, develop colitis, and show increased intestinal epithelium permeability to bacteria [26]. 

Many intestinal diseases are associated with some degree of altered mucin expression. CD patients show decreased MUC1 and MUC4 levels in the ileum, while MUC2, MUC5AC, MUC5B, MUC6, and MUC7 are undetectable in lesions [27]. UC patients also show decreased MUC2 expression as well as lower O-glycosylation and sulfation [28–30].

Other protein constituents of intestinal mucus are trefoil peptide-3 (TFF-3), resistin-like molecule-*β* (RELM-*β*), and protein-binding Fc-*γ* (Fcgbp). 

TFF-3 belongs to the family of trefoil factors. It can be found inside goblet cells as a monomer (6,6 kDa) and dimer (13 kDa) and is the second-most abundant molecule secreted by intestinal cells. TFF-3 protects the epithelium, repairing damage, facilitating cell migration, and blocking apoptosis. Furthermore, when TFF-3 is coexpressed with MUC2 protein, mucus viscosity increases, probably because TFF-3 can bind to MUC2 Ddomains [31, 32]. TFF-3^−/−^ mice are more susceptible to developing a DSS-induced colitis than wild-type mice, while animals overexpressing this protein are more resistant to damage and intestinal ulceration [22, 33]. In UC patients, serum TFF-3 levels increase with disease activity and decrease upon corticosteroid treatment [34]. However, increased serum TFF-3 levels do not reflect intestinal mucosa content, as goblet cells of UC patients have a low protein content, which may be a sign of cell necrosis [35].

RELM-*β* belongs to the family of resistins and is a cysteine-rich protein secreted by goblet cells, mainly as a homodimer [36]. RELM-*β* induces MUC2 secretion, which increases upon cell exposure to bacteria and parasites [37, 38]. Fcgbp is a protein also secreted by goblet cells and combines, through disulfide bonds, with MUC2 and TFF-3, allowing for greater mucus layer cohesion and viscosity [39]. Fcgbp binds to the Fc portion of IgG molecules, and, during an inflammatory reaction, the complex is fixed to the mucus layer, enhancing bacteria opsonization [40, 41].

RELM-*β* and Fcgbp expression and content have not yet been studied in IBD patients, and the role of these proteins in disease remains unknown.

### 2.2. Epithelium as a Second Line of Intestinal Defense

Epithelial cells are organized into villi and intestinal crypts (Lieberkühn crypts). Small intestine villi are longer and crypts shallower than their counterparts in the colon. The intestinal epithelium is composed of a monolayer of fast-replicating, polarized cells of three types: enterocytes, goblet cells, and enteroendocrine cells. All are bound together through tight junctions that separate the body from intestinal lumen components. Enterocytes are the most abundant cells. These cells allow for nutrient absorption and secret microbicidal proteins, such as defensins and cathelicidins [42]. Goblet cells secrete intestinal mucus (described in Section  2.1), and enteroendocrine cells produce hormones, such as serotonin, substance P, and secretin. These cells are located in the crypt base along with Paneth cells, which secrete *α*-defensins, phospholipase A2 (PLA2), lysozymes, and other antimicrobial peptides [43, 44]. 

Other cells involved in intestinal immunity are M cells, which are located in the Payer's patches. They are particularly abundant in lymphoid follicles of the small intestine, an area scarce in goblet cells and lacking a mucus layer. The function of M cells function is to translocate and present microbiota-derived antigens to dendritic cells and macrophages. 

Phagocytic cells express receptors that sense pathogen-associated molecular patterns (PAMPs), allowing for differentiation between self- and non-self-molecules. The most characteristic PAMPs in the intestinal lumen are cell wall components of gram-positive and -negative bacteria, such as proteins, carbohydrates, and lipids with repetitive arrangements, flagella subunits, bacterial unmethylated CpG dinucleotide DNA, and viral double-stranded RNA. Pathogen-recognition receptors (PRRs) are expressed in epithelial cells, macrophages, neutrophils, and dendritic cells, activating phagocytosis, chemotaxis, and effector molecule production. These processes contribute to a late innate or adaptive immune response. 

Among intestinal PRRs, the most important are the membrane Toll-like receptor (TLRs) and cytosolic nucleotide oligomerization domain receptor (NOD). Mammalian TLRs comprise a highly evolutionarily conserved recognition and signaling system. In humans, ten functional TLRs recognize certain PAMPs and activate the NF-*κ*B-signaling pathway to induce proinflammatory genes. Some mammalian TLRs are distributed on the cell surface (TLR2, TLR4, and TLR5), while others are found in the endosomal membrane (TLR3, TLR7, and TLR9) (Figure 1(d)) [45–48].

Intestinal epithelial cells are in contact with nutrient and commensal bacteria antigens that may or may not activate the inflammatory pathways; the epithelial TLRs provide special control mechanisms for this process. Although data relating to TLR expression and compartmentalization is contradictory, there are reports indicating that TLRs are exclusively expressed in intestinal crypts, and not in villi where greater contact with luminal antigens occurs [49–52]. TLR4 sequestration in the Golgi apparatus and low expression of the MD2 coreceptor in epithelial cells may represent other mechanisms that prevent TLRs from activating proinflammatory signals after contact with intestinal antigens [51, 53, 54]. TLR9, located in the plasma membrane of intestinal epithelial cells, activates a response dependent on apical or basal location; that is, it activates a tolerogenic response or NF-*κ*B-dependent proinflammatory pathway in the apical or basal membrane, respectively [55].

NOD receptors are capable of binding microbial products. The NOD1 isoform recognizes gram-negative bacteria PAMPs, while NOD2 detects muramyl dipeptide present in proteoglycans of gram-positive and -negative bacteria. These receptors, as TLRs, are expressed in cells continuously exposed to bacteria, mainly epithelial cells, macrophages, and dendritic cells [56, 57]. They activate NF-*κ*B and MAPK pathways synergizing with TLRs in cytokine production [58, 59]. However, NOD2 has been also described as a negative regulator of TLR2-mediated IL-12 secretion [60, 61]. Some *Nod2* mutations have been described in CD patients [62], associated with decreased defensin secretion by ileal mucosa Paneth cells [63].

Several changes in the intestinal epithelium of UC patients have been reported, such as epithelial cell proportions, goblet cell content, and reticulum stress response. Goblet cells represent 55% of total cells in the colonic epithelium of control individuals, compared to 33% in active UC patients. Moreover, goblet cells are diminished primarily in the upper third of intestinal crypt in UC, representing 48% and 27% of total cells in this region in control and UC patients, respectively [64]. Paneth cells are usually found in the small intestine; however, UC patients have ectopic Paneth cells in the colon that secrete aberrant antimicrobial proteins, such as *β*-defensins and cathelicidins [65–67].

Atypical goblet cell proteins have also been identified in UC patients, such as an immature mucin not correctly glycosylated as well as an empty apical theca [68]. Moreover, endoplasmic reticulum stress levels in UC intestinal mucosa are higher than in controls [68, 69], potentially altering cytosolic calcium levels and NF-*κ*B pathway activation, thereby increasing epithelial permeability [70–72]. 

### 2.3. *Lamina propria* as a Third Line of Intestinal Defense

Immediately below the epithelial layer is the *lamina propria*, containing innate and immune cells, such as macrophages, neutrophils, polymorphonuclear, dendritic, and natural killer cells, and lymphocytes, respectively. In this paper we will focus on the cells involved in innate immune responses and the changes occurring in UC patients. 

*Dendritic cells* are myeloid phagocytic cells that migrate from bone marrow to various tissues through the bloodstream. In the intestinal mucosa, dendritic cells are mainly located in the *lamina propria* and Peyer's patches, reaching the epithelium sending prolongations without disrupting epithelial integrity phagocyting luminal antigens independent from M cells. Intraepithelial localization of dendritic cells responds to fractalkine (CX3CL1), expressed in the epithelial cell plasma membrane that binds to CX3CR1, expressed on its plasma membrane [73–75].

Besides their phagocytic capacity, dendritic cells capture extracellular fluid through macropinocytosis and participate in T-cell antigen presentation in lymph nodes, initiating adaptive immune responses and comprising a central link between both immune responses [76–80].

IBD patients have atypical dendritic cell phenotypes in terms of both maturation state and anatomical localization [81]. Dendritic cells accumulate in inflamed intestinal tissue, possibly as a result of increased expression of the chemokine CCL20. This molecule is regulated by NF-*κ*B and induces dendritic and T-cell recruitment. Furthermore, colonic CD11c+ dendritic cells from UC and CD patients express higher TLR2, TLR4, and CD40 levels as compared to remission patients or healthy individuals [82]. In IBD patients, dendritic cells promote a robust recognition of bacterial products that might cause immune response activation to commensal bacteria, provoking a loss of intestinal tolerance. 

*Macrophages* are long-lived myeloid-derived cells and, along with neutrophils and dendritic cells, are central phagocytic cells of the immune system. Macrophages degrade captured pathogens through lysosomal enzymes present in phagosomes and phagolysosomes. Degraded peptide products migrate, combined with MHC-II molecules, to the cell surface. There they initiate antigen presentation, integrating with dendritic cells, for both innate and adaptive immunity.

Monocytes are an immature type of macrophage, found in peripheral blood and migrating to tissues, maturing if necessary. Monocyte destiny is dependent on chemoattractant cytokines, such as IL-8 and TGF-*β*, constitutively produced by intestinal epithelial cells and mast cells [83]. Macrophages express several PRRs, whose content is increased upon cell activation [84, 85]. Furthermore, intestinal macrophages have tolerogenic features, with low expression of PRRs and other surface proteins, such as CD14 and coactivator molecules CD80 and CD86 [86]. In addition, intestinal macrophages secrete limited amounts of IL-1, IL-6, and IL-8, likely due to reduced MyD88 expression, an important adaptor molecule in TLR/IL-1-dependent NF-*κ*B activation [87]. However, these cells express high levels of IL-1 precursor when exposed to microbiota, indicating that they actively contribute to mucosal tolerance [88].

Intestinal macrophages, as well as dendritic cells, localize in *lamina propria* and are dependent on fractalkine (CX3CL1) content [89]. Mice deficient in fractalkine receptor (CX3CR1^−/−^) are more susceptible to *Salmonella typhimurium* infection, possibly due to lower capacity for phagocyte recruitment to *lamina propria* [90].

Intestinal macrophages from IBD patients have lost the ability to maintain tolerance, mainly through increased surface CD14 content [91] and NF-*κ*B transcription pathway activity [87, 92], which might induce increased peripheral macrophage recruitment. 

*Granulocytes* are myeloid-derived cells with a short half-life. These cells are rich in cytoplasmic granules and are considered polymorphonuclear leukocytes due to an irregular nucleus. Three types of granulocytes have been described—neutrophils, basophils, and eosinophils—based on different granule coloration properties.

Neutrophils are the most abundant leukocytes in blood, accounting for 60% of the total in noninflammatory states. A significant role in innate immune response has been attributed to neutrophils, as robust phagocytic activity is driven by TLR and/or NOD stimulation. In addition, activated neutrophils secrete antimicrobial molecules, reactive oxygen species, inflammatory cytokines, and chemokines that recruit dendritic cells and macrophages into the mucosa [93, 94]. These secreted products may adhere to fibrillar networks secreted by neutrophils, composed mainly of DNA (neutrophil extracellular traps), allowing for interaction with bacterial components [95].

Neutrophil-specific chemoattractant cytokines are mainly produced by epithelial cells, such as IL-8 and hepoxilin A3 [96–98], secreted through the apical and basal cell poles, respectively. Concentration gradients of cytokines cause neutrophil transepithelial migration. In line with this, IL-8 secretion is elevated in damaged epithelium from IBD patients, resulting in augmented neutrophil recruitment and exacerbated immune response in the intestinal mucosa [99]. Another important protein is calprotectin, which is present both in serum and faeces. Its concentration increases considerably during inflammatory conditions, including IBD. Faecal calprotectin is sometimes used as a screening test for patients who may require further invasive diagnostics. Furthermore, there have been reports on the use of calprotectin assays in monitoring treatment of pediatric and adult UC and CD patients [100].

The functional characteristics of eosinophils and basophils have not yet completely studied. These cells secrete proinflammatory cytokines and proteins important in host defense against parasites [101–103]. IBD patients show increased expression of eosinophil- and basophil-associated chemokines, such as eotaxin and MCP-3, suggesting that these cells might have an important role in the pathophysiology of these diseases [104, 105]. This is reflected in the increased eosinophilic cationic protein content in faeces of IBD patients [106], increased eosinophilic granule protein concentration in intestinal fluid and peripheral blood of these patients [107, 108], and increased eosinophil content in intestinal mucosa of active CD patients [109]. 

*Mast cells* protect mucosal tissue against pathogens and parasitic worms. They have an important role in allergic response. Mast cell cytoplasmic granules contain large amounts of histamine, tryptase, prostaglandin PGD2, leukotriene LTC4, and eotaxin [110]. However, intestinal epithelium permeability might be affected by tryptase derived from mast cells, shown to modulate intercellular tight junctions through the activation of the PAR-2 receptor [111]. 

The role of mast cells in IBD is still not completely understood, and reported information is controversial. Increased mast cell numbers in the intestinal mucosa of active patients have been reported [112–114]. However, no difference in cell content was detected in control and inactive patients [115, 116] although damaged mucosal tissue may have fewer mast cells [117], possibly resulting in granule content liberation and extracellular matrix damage.

Moreover, mast cell activation and intestinal epithelium permeability are dependent on serum levels of stress hormones (cortisol and ACTH). Mast cell activation is increased in inactive IBD patients subjected to a cold pressor test. Although the mechanism involved in mast cell activation is not completely understood, an association with c-kit receptor content and mast cell maturation has been described [118–122].

*Intraepithelial *γ*/*δ* T lymphocytes* are lymphoid cells with a granular cytoplasm, located in the epithelium of various organs, especially intestine, skin, lung, and reproductive tract. *γ*/*δ* T lymphocytes have a limited TCR repertoire and are activated by a limited variety of antigens, recognizing mainly self-antigens expressed by stressed epithelial cells [123]. The most abundant intestinal population is the intraepithelial lymphocytes (IELs) V*γ*5+, whose expression depends on thymic maturation driven by IL-15 [124]. When cells are activated, inflammation is modulated, and wounds are efficiently healed. Tissue homeostasis is achieved independently of constant exposure to environmental changes. *γ*/*δ* T lymphocytes produce keratinocyte growth factor, which stimulates proliferation and differentiation of epithelial cells [125] as well as chemokines involved in T reg recruitment into the *lamina propria* [126]. 

In IBD patients, IEL activation increases, resulting in elevated production of IFN-*γ*, TNF-*α*, and IL-2, associated with increased IL-23 [127] and fractalkine content, as well as CXC3R1 in polymorphonuclear lymphocytes in intestinal mucosa [89–128]. Adoptive transfer of ILEs into mice with chemically induced colitis lacking intraepithelial lymphocytes *γ*/*δ* promoted a decrease in IFN-*γ* and TNF-*α* and an increase in TGF-*β* levels [129], suggesting a critical role of these cells in mucosal tolerance.

*Natural killer (NK) cells* are lymphoid cells with a granular cytoplasm. These cells do not respond to a specific antigen; however, they are able to recognize aberrant cells, such as those infected with viruses and other intracellular pathogens [130]. The activation of NK cells induces cytoplasmic granule exocytosis, liberating perforins and granzymes that promote abnormal cell apoptosis. NK cells recognize specific cell surface death receptor ligands in target cells, activating caspase-dependent apoptosis [131]. 

The role of NK cells in IBD pathogenesis is still not well understood. However, human intestinal mucosa of IBD patients show increased cytotoxic activity and elevated NK cell counts in the *lamina propria* [132, 133]. These findings could be a consequence of imbalanced cytokine and growth factor content in intestinal mucosa of IBD patients. Crucial molecules in NK cell development, such as IL-15, IL-21, and IL-23, and their cognate receptors, are elevated in the intestinal mucosa of UC patients. IL-15 and IL-21 promote LT, LB, and NK cell differentiation, while IL-23 controls memory T-cell mechanisms and promotes Th17 cell proliferation and survival. Therefore, imbalanced cytokine content in UC patients may, in part, cause the activation of NK cells. Recent studies have evaluated an intervention using IL-21R as a therapeutic target in IBD patients, with promising results [134–137].

## 3. The Role of the IL-33/ST2 System in Ulcerative Colitis

In recent years, scientific interest in the significance of the IL-33/ST2 system in IBD physiopathology has grown. Increased expression of IL-33 and its receptor (ST2) has been reported in the intestinal mucosa of UC patients [1, 138–140]. In addition, expression of the soluble ST2 isoform (sST2) in serum is correlated with intestinal mucosa ST2 levels as well as with disease severity, behaving as a potential UC activity biomarker [2].

IL-33 belongs to the IL-1 superfamily, along with IL-1*α*, IL-1*β*, and IL-18, whose genes are located on human chromosome 9p24.1 [141]. IL-33 is distributed in the cytoplasm and nucleus of endothelial cells, fibroblasts, adipocytes, smooth muscle cells, macrophages, and dendritic cells [139, 141–143], while ST2 is mainly expressed in mast cells, macrophages, and Th2 lymphocytes [139, 141–144].

The ST2 gene belongs to the IL-1/TLRs receptor superfamily and is located in human chromosome 2q12. Transcription *ST2* gene products comprise four protein isoforms, which are generated by alternative splicing: ST2L, sST2, ST2LV, and ST2V [145]. The most abundant isoform is transmembrane ST2L and soluble sST2, which is identical to the extracellular ST2L domain and nine additional amino acids. sST2 acts as a decoy receptor, sequestering IL-33 and inhibiting binding to ST2L [146, 147]. Furthermore, ST2V is similar to ST2L, lacking the third immunoglobulin domain. ST2LV has no transmembrane domain [148, 149].

**Figure 2:**
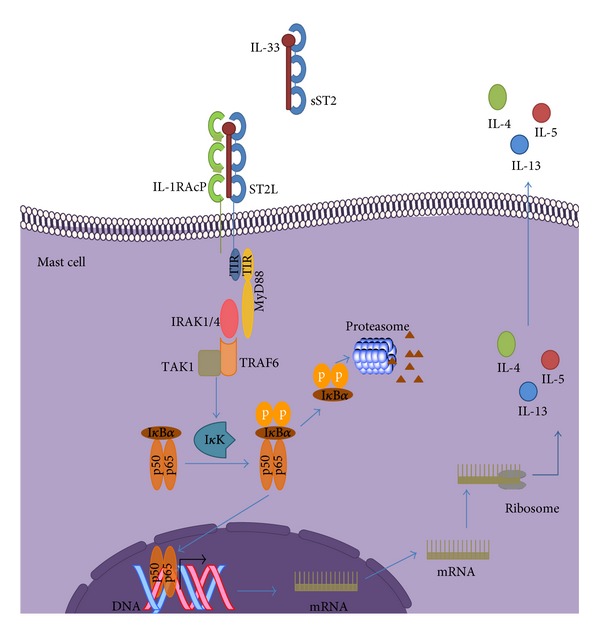
IL-33/ST2 system. IL-33 recognition by ST2L promotes receptor dimerization with IL1RAcP and recruitment of receptor complex components, MyD88, TRAF6, and IRAK1-4, to intracellular TIR domain. Furthermore, protein phosphorylation favors NF-*κ*B and MAPK pathway activation. When sST2 is translated and secreted to the extracellular media, it sequesters IL-33 and inhibits binding to ST2L as a decoy receptor.

IL-33 recognition by ST2L promotes receptor dimerization with the IL-1 receptor accessory protein (IL1RAcP) [150]. The intracellular TIR domain of receptor complex components is subjected to phosphorylation, favoring MyD88, TRAF6, and IRAK1-4 recruitment and subsequently activating NF-*κ*B and MAPK pathways [141]. ST2 can also dimerize with a second IL-1R family coreceptor, SIGIRR, which negatively regulates the IL-33/ST2-signaling pathway. However, the molecular mechanism and its pathophysiologic relevance are not yet completely understood [151] (Figure 2). 

In addition, studies have demonstrated that IL-33 migrates into the nucleus to sequester NF-*κ*B and inhibits transcriptional activity, pointing to a possible mechanism as a transcription factor in epithelial cells [152]. Therefore, IL-33 has a dual function as a pro- and anti-inflammatory cytokine. 

Unlike IL-1 and IL-18, IL-33 activity does not require caspase-1 processing. Rather, the processed IL-33 is less active than the complete form. This might be a regulatory mechanism to avoid excessive inflammatory response when IL-33 is released from the cell [153]. A similarity with other members of this cytokine superfamily is the absence of a secretion signal for the classical endoplasmic reticulum/Golgi apparatus pathway [141]. Therefore, as with IL-1*α*, IL-33 is considered a necroalarmin, released into the extracellular medium during necrosis in an inflammatory response. Recent studies show that IL-33 also exports the nucleus through the nuclear pore complex and is stored in cytoplasmic vesicles; in addition, it is secreted as a completed form by stressed cells. Thus, IL-33 is a paracrine mechanosensitive alarmin [154]. 

## 4. Role of IL-33 in the Innate Immune Response

Given the reports of an imbalanced IL-33/ST2 system in UC patients, it would be instructive to catalogue effects of this cytokine along with the different innate immune barriers and evaluate possible consequences. Because epithelial cells and myofibroblasts are the main IL-33 sources in the intestinal mucosa, and the main target cells are found in the *lamina propria*, this section will focus on the effects of IL-33 on epithelial and *lamina propria* cells and impact on the quality of secreted mucus (Table 1).

*IL-33/ST2 effect on intestinal epithelium:* deregulated mucin expression in IBD patients might be due to the cytokine imbalance that characterizes these diseases. Th1 (IL-2, IL-12, IFN-*γ*, and TNF-*α*) and Th2 cytokines (IL-4, IL-5, and IL-13) are upregulated in CD and UC patients, respectively. These molecules stimulate various transcription factor pathways, such as JAK/STAT and NF-*κ*B, and induce mucin secretion [155–157]. However, IL-33 might also have a mucosecretagogue activity, as IL-33-treated mice show increased mucin content in intestinal goblet cells [141]. The molecular mechanism of this process remains unknown, but it likely involves NF-*κ*B activation, as the MUC2 promoter contains transcription factor-binding sites [158].

The mechanism by which IL-33 regulates secretory products of intestinal goblet cells, in particular for TFF-3 and RELM-*β*, has not been reported. Although IL-33 has been shown to modulate TFF-2 and RELM-*α* production by pulmonary cells, no information regarding its effect on intestinal tissue is available. In pulmonary-infected mice, TFF-2 is necessary for rapid IL-33 production by respiratory epithelium, alveolar macrophages, and dendritic cells [159]. Moreover, IL-33 administration to *Pneumocystis-*infected mice increases RELM-*α* levels [160]. However, the relationship of IL-33 and TFF-3 with RELM-*β* molecules has not been studied in UC patients, where an inverse content of IL-33 and TFF-3 has been observed in intestinal mucosa [35]. 

*IL-33/STS2 effect on lamina propria: lamina propria* cells are IL-33 sources, although not as important as epithelial cells. *In vivo* studies have demonstrated that LPS-stimulated murine macrophages increase IL-33 transcript and protein levels in the extracellular medium [161]. Furthermore, IL-33^−/−^ macrophages stimulated with LPS secrete lower levels of IL-6 and TNF-*α* than wild-type macrophages. Moreover, counteracting the effects of IL-33 with a specific antibody in wild-type macrophages partially reverted LPS-induced cytokine secretion, as compared to IL-33^−/−^ cells. This finding can be explained by the fact that secreted IL-33, but not the nuclear effects of IL33, were inhibited [162].

IL-33 synergizes chemoattractant effects of macrophage cytokines, such as TNF, IL-1*β*, CXCL1, and CCL3, on neutrophil recruitment. In addition, IL-33 may directly regulate neutrophil mobilization [163]. 

Mast cells and basophils are the main IL-33 targets in *lamina propria*, as surface ST2 expression is increased upon IL-33 stimulation. Both cells secrete Th2 cytokines and chemokines when stimulated by IL-33, and although it does not induce direct degranulation, it synergistically increases IgE-mediated degranulation. Moreover, IL-33 induces eotaxin-mediated migration of mast cells [164–167].

Peripheral eosinophils express low ST2L levels at the cell surface although transcript and intracellular ST2 content has been detected in basal conditions. Furthermore, eosinophil stimulation with IL-33 induces peroxide and IL-8 as well as IL-3, IL-5, and GM-CSF production. In addition, like basophils, eosinophils increase CD11b expression upon IL-33 stimulation; however, exotoxin-mediated migration is not influenced by the cytokine [168, 169].

As a final point, dendritic cells also express ST2 and, when stimulated with IL-33, IL-6, CD86, and MHC-II, expression increases [170] and allows naïve T cells to differentiate into Th2 cells. This does not occur with direct stimulation of T cells as they lack surface expression of ST2 [171, 172].

## 5. Projections

Although in recent years there has been great advancement in studies of the role of IL-33/ST2 in epithelium and the potential effects of its imbalanced expression on tissue function, there are still many questions regarding the impact of this system on IBD pathologies. 

For many years, IL-33 had been thought to function exclusively as an alarmin although recent studies reported that IL-33 may be also secreted. However, the mechanisms involved in this process remain unknown. Moreover, the role of ST2 in pathology has not been described completely. Increased expression of the soluble ST2 isoform might be turned on as a mechanism to compensate for the proinflammatory effect mediated by IL-33. On the other hand, increased sST2 might induce mucosal damage and consequently inflammation. 

There are numerous intestinal diseases that show inflammatory conditions; however, the role of the IL-33/ST2 system has not been resolved. It would be interesting to understand the impact of this inflammatory pathway on inflammatory conditions of the intestine and assess, if possible, strategies to solve this imbalance towards a reduction of inflammation.

## Figures and Tables

**Table 1:** Functions of intestinal innate immune cells, role in IBD, and response to IL-33 stimulation.

Cell	Function	Role in IBD	IL-33 effect
Goblet cell	Secretion of mucin, TFF3 and RELM-*β*.	Aberrant accumulation of mucin. Low TFF3 secretion.	Mucosecretagogue activity.
Macrophage	Phagocytosis. Antigen presentation. Intestinal antigenic tolerance.	Loss of tolerance. Increased CD14 and NF-*κ*B expression.	Secretion of TNF, IL-1*β*, CXCL1, and CCL3.
Basophil mast cell	Defense against parasites. Allergic response.	Controversial.	Increased expression of surface ST2. Th2 cytokine secretion. Synergistically increases the IgE-mediated degranulation.
Eosinophil	Defense against parasites. Allergic response.	Higher eosinophilic protein content in feces and intestinal fluid.	Production of superoxide, IL-3, IL-5, IL-8, and GM-CSF.
Dendritic cell	Antigen presentation. Phagocytosis. Macropinocytosis.	Accumulation in inflamed area. Increased expression of TLR2, TLR4, and CD40.	Increases expression of MCH-II, CD86, and IL-6. Th2 differentiation.
